# Environmental DNA analysis of river herring in Chesapeake Bay: A powerful tool for monitoring threatened keystone species

**DOI:** 10.1371/journal.pone.0205578

**Published:** 2018-11-01

**Authors:** Louis V. Plough, Matthew B. Ogburn, Catherine L. Fitzgerald, Rose Geranio, Gabriella A. Marafino, Kimberly D. Richie

**Affiliations:** 1 Horn Point Laboratory, University of Maryland Center for Environmental Science, Cambridge, Maryland, United States of America; 2 Smithsonian Environmental Research Center, Edgewater, Maryland, United States of America; University of Hyogo, JAPAN

## Abstract

Environmental DNA (eDNA) sampling has emerged as a powerful tool to detect and quantify species abundance in aquatic environments. However, relatively few studies have compared the performance of eDNA-based abundance estimates to traditional catch or survey approaches in the field. Here, we have developed and field-tested a qPCR assay to detect eDNA from alewife and blueback herring (collectively known as ‘river herring’), comparing eDNA-based presence and abundance data to traditional methods of quantification (ichthyoplankton sampling and adult observations). Overall, the qPCR assay showed very high target specificity in lab trials, and was successful in detecting river herring for 11/12 Chesapeake Bay tributaries in spring 2015 and 2016, with 106 out of 445 samples exhibiting positive eDNA hits. We found a strong correlation between eDNA abundance and ichthyoplankton count data (Spearman’s Rho = 0.52), and Phi-tests (correlation of presence/absence data) showed higher correlation between eDNA and ichthyoplankton data (Phi = 0.45) than adult data (Phi = 0.35). Detection probability was significantly lower on western vs. eastern shore tributaries of Chesapeake Bay, and blueback herring and alewife were more likely detected on the western and eastern shores, respectively. Temporal patterns of eDNA abundance over the spring spawning season revealed that alewife were present in high abundances weeks ahead of blueback herring, which aligns with known differences in spawning behavior of the species. In summary, the eDNA abundance data corresponded well to other field methods and has great potential to assist future monitoring efforts of river herring abundance and habitat use.

## Introduction

Accurate information on the abundance and spatial distribution of aquatic species is essential for understanding their ecology and for their management in increasingly human-impacted environments. However, capture-based sampling and tracking of mobile species, such as fish, are complicated by their movements or migration, which can vary seasonally, daily, or in response to environmental change [1–5]. For invasive, threatened, or rare species that are at low densities, accurate estimates of abundance may be even more challenging due to low probability of encounter in the environment [6–10]. Traditional approaches to assess fish abundance involve relatively invasive capture methods such as electrofishing or net collection, which vary in their efficiency and precision (e.g. [11–16]), may be challenging to implement in certain environments (e.g. [17]), and can be harmful to threatened species [18–19]. Other visual or acoustic-based methods to survey and enumerate fish, such as video capture or active sonar, present alternative, less invasive approaches that can be quite effective and quantitative [20–27], but may also be expensive and laborious to deploy over large spatial scales.

Environmental DNA (eDNA) sampling or surveillance has recently emerged as a powerful, non-invasive alternative to capture-based techniques to detect the presence/absence or abundance of species in their environment (e.g. [28–31]). eDNA sampling is based on the premise that species (aquatic and terrestrial) leave behind DNA in the environment through the sloughing of cells, excretion of urine/feces, or release of gametes, which can be detected in soil or water (after DNA extraction) via highly sensitive molecular approaches like quantitative PCR [30,32]. eDNA analysis appears to be particularly well-suited to detect rare or elusive taxa in aquatic environments because of the high sensitivity of the assays (detections down to a few DNA copies) and the relatively simple sampling protocol (~0.5–3 liters sampled from surface waters), which allows widespread sampling across a range of field conditions that can be difficult for traditional survey approaches such as electrofishing [33–35]. Initially developed for detection of species in freshwater environments (e.g. [33, 35, 36–38]), eDNA analysis has now been applied extensively in other systems, including marine and estuarine environments [39–41], detecting and quantifying a number of vertebrate and invertebrate species to answer ecological and conservation questions.

As the field of eDNA analysis has grown and matured, significant advances have been made in understanding the effect of various environmental and technical factors on the detection of eDNA, as well as the potential for eDNA abundance data to predict actual abundances in the environment [31,42]. For example, lab or mesocosm-based studies have explored the role of temperature on both degradation of eDNA and eDNA shedding rates (e.g. [43–46]), as well as how light, time, and flow impact eDNA shedding and detection [47–49]. A variety of preservation and sampling approaches have also been tested (e.g. [50]) and detailed analyses of the effect of filter material and pore size on eDNA abundance has been conducted [51–52]. Progress has also been made examining the association between eDNA abundance and actual fish abundances or biomass. In laboratory, mesocosm, and field studies, eDNA and biomass often show strong, positive associations (e.g. [33, 36, 44]) using either qPCR (copy number) or high-throughput metabarcoding data [40, 53]; however, the strength of these associations vary significantly, especially in the field [42]. Biomass appears to be a better predictor than abundance when available, but some debate still exists about how well eDNA data can match actual density or biomass estimates from more traditional catch-based survey approaches (e.g. [54]) and environmental factors are known to have a major effect on eDNA detection probabilities in the field (e.g. [46–47]). Models of detection probability that incorporate expected eDNA degradation (decay) rates and transport under a range of environmental conditions informed by experimental data may improve the accuracy of abundance inferences from eDNA (e.g. [44, 52]), but more work is needed in this area. Before eDNA can become more widely integrated into conservation or management programs, additional studies comparing eDNA abundance with traditional fisheries data are needed.

In this study, we developed and tested an eDNA assay to detect and quantify the abundance of two anadromous fish species, alewife *Alosa psuedoharengus* and blueback herring *Alosa aestivalis*, collectively known as river herring, in the Chesapeake Bay watershed. Ranging from Newfoundland to South Carolina (alewife), and the Gulf of St. Lawrence to the St. Johns River in Florida (blueback herring), river herring enter freshwater environments in spring to spawn, and play important roles in coastal food webs as agents of nutrient transfer between marine and freshwater environments and as prey for coastal birds and fishes [55–57]. One of the oldest fisheries in North America, river herring landings have declined sharply since the 1970’s [58, 59] due to loss of spawning habitat, overfishing, and degradation of water quality. River herring are currently considered species of concern across their range [59–62]. The mid-Atlantic stocks of river herring, which include Chesapeake Bay spawning runs, are of particular conservation concern based on declining trends in fishery landings [63] and apparent susceptibility to by-catch in the Atlantic herring fishery [64]. While long-term stock status information based on run count data is available in some regions, it is particularly lacking in Chesapeake Bay [58]. Monitoring efforts in the Chesapeake Region have recently been established using Dual-frequency Identification Sonar (DIDSON) and electronic fish counters, the former showing promise for accurately quantifying run strength in turbid, free-flowing streams [27] and the latter showing promise where fish passage is restricted to a narrow fish passage structure (Alan Weaver, personal communication). However, these approaches can be costly to install and maintain and only provide information for the particular locations where they are installed. Alternative methods to estimate presence or abundance of river herring, such as eDNA sampling, have been proposed as potentially cost-saving approaches to monitor the abundance of river herring over a wider geographic scale in the Mid-Atlantic.

**Fig 1:**
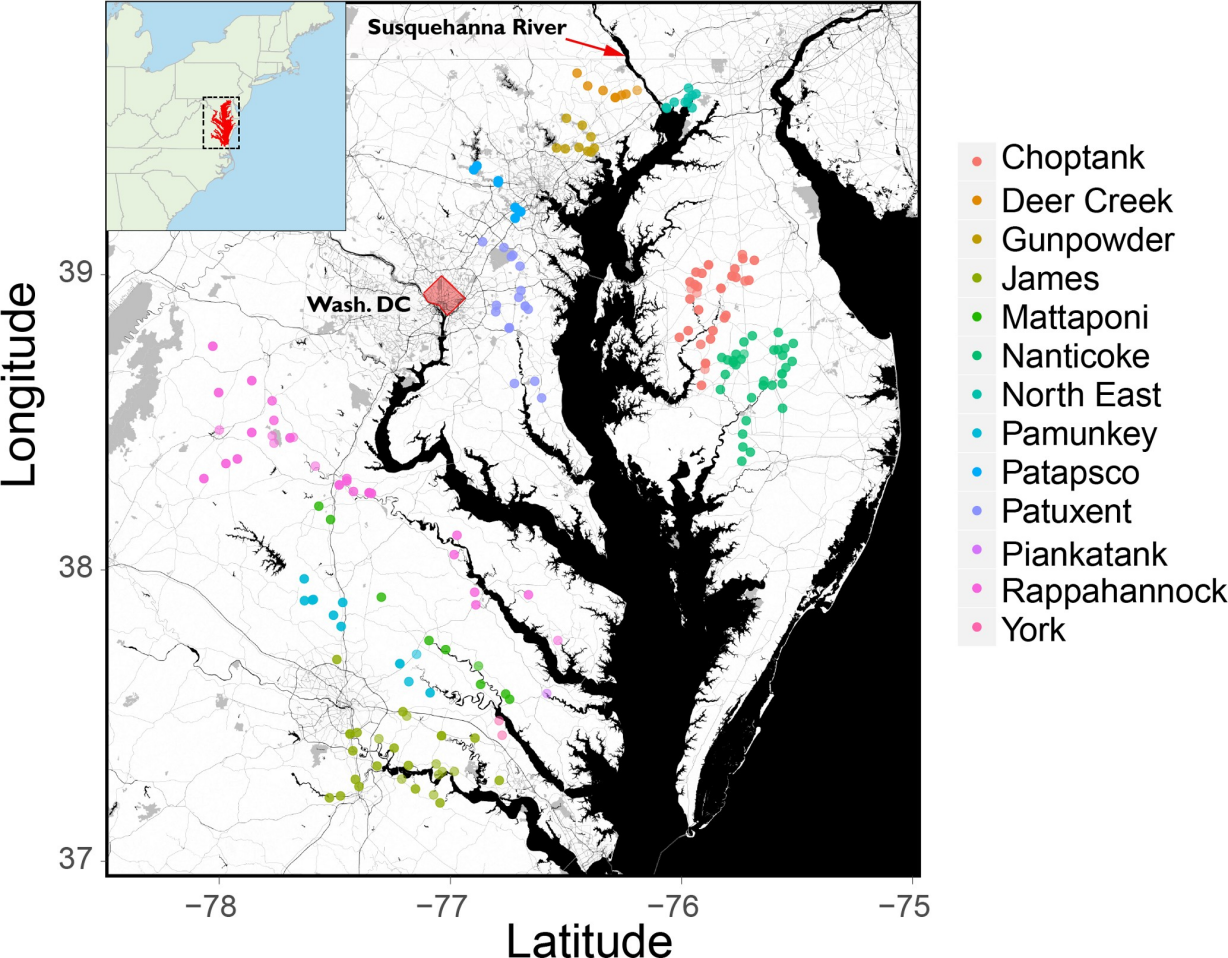
Map of eDNA and field sampling locations in Chesapeake Bay. Inset shows the focus region (Chesapeake Bay in Red) within the United States of America.

Here, we present results on the development of a qPCR assay for river herring and its performance over two years of eDNA field sampling (spring 2015, 2016) across a number of major tributaries and rivers in Chesapeake Bay (Fig 1). We also examine associations between abundance estimates based on eDNA data vs. traditional rapid assessment methods (visual/cast net observation of adults and ichthyoplankton surveys), as well as patterns of eDNA abundance across major tributaries of the Chesapeake Bay and over the spawning season.

## Materials and methods

### Assay development

Publicly available mitochondrial DNA sequences from river herring and a number of phylogenetically similar fish species (e.g. other shad, alosids and clupeids) were collected from NCBI Genbank and aligned in AliView v.1.18 [65] to examine the suitability of different loci for designing a qPCR assay. Significant numbers of sequences were available for cytochrome oxidase 1 (hereafter CO1), cytochrome B, NADH2, and 18SrRNA among others, but only CO1 contained regions with sufficient numbers of diagnostic nucleotide differences between river herring and other closely related alosids for probe design. In particular, relatively low sequence divergence was found between American shad (*Alosa sapidissima*) and river herring for many of the loci screened, which reflects their relatively close evolutionary history (e.g. [66]). Hickory shad *Alosa mediocris* is even more closely related to river herring, but fewer mitochondrial sequences were publicly available, and none for CO1 at the time of assay design. To supplement alignments of CO1 for design of the probe/assay, unpublished sequences for hickory shad and a number of other Chesapeake Bay fishes (especially clupeids) were added from the Smithsonian’s Chesapeake Bay Barcode Initiative (GenBank accession numbers MH570218-MH570250; sequences in S1 File). In total, 68 sequences from 12 species including all alosine fish present in Chesapeake Bay were used in the alignment, with a minimum of ~600 bp shared among sequences (S1 File). A ~50 bp region of CO1 exhibiting ~8–10 sequence differences between river herring and American or hickory shad (but no polymorphism between alewife and blueback herring) was targeted for probe design. Because of the very high sequence similarity between alewife and blueback herring, the probe and primer set was designed to amplify both species. Identification to species (alewife or blueback herring) was performed after positive eDNA (qPCR) detection via Sanger sequencing (see below).

### Details of the qPCR assay

Molecular beacon probes (e.g. [67]) and PCR primers were designed in the OligoArchitect software (Sigma Aldrich) targeting a ~50 bp region within CO1 and requiring that amplicons be 200 bp or smaller. We chose to design a molecular beacon assay to provide better target specificity and reduce off-target amplification or hybridization to highly similar shad DNA. Four primer/probe sets met the software’s design threshold and were ordered from Integrated DNA Technologies (IDT, Coralville, IA), but only one produced consistent amplification, low background, and minimal off-target amplification in initial testing with fin-clip DNA samples. The beacon was dual-labeled with a fluorophore (FAM) and quencher (IOWABlack; see Table 1 for primer and probe information). Quantitative PCR was performed on the BioRad CFX 96 qPCR instrument (Biorad, Hercules CA) in 20 μl volumes with 10 μl of BioRad 2X SSO advanced universal probe super mix, 0.7 μl of each primer and probe (10μM), 3.9 μl of water, and 4 μl of DNA. PCR cycle conditions were as follows: 95°C for 45 seconds followed by 44 cycles of 95°C for 5 seconds, 60°C for 15 seconds, and 72°C for 10 seconds, with a plate read after each cycle. Cycle threshold (Cq) values for each sample were determined with the BioRad CFX Manager software (v3.1) using a single-point threshold set at 100 RFU and the baseline substitution curve fit for drift correction.

**Table 1:** qPCR primers and PCR conditions.

Primer name	Sequence	Cycle conditions^2^
CO1_RH-F	ATGAGCTTCTGACTACTT	1. 95C for 45 sec
		2. 95C for 5 sec
CO1_RH-R	GATAGTTAGATCGACGGA	3. 60C for 15 sec
		4. 2 C for 10 sec
Beacon_RH^1^	CGCGATCGGATGAACAGTCTACCCGCCCTTGATCGCG	5.Go to step 2 for 39* cycles

^1^Beacon synthesized as 100 nm IDT ‘PrimeTime’ probe, labeled 5' 6-FAM / 3' IBFQ.

^2^Plate read on CFX96 after ever cycle See methods for PCR/probe reagents.

*Forty-five cycles were run for initial assay testing, but amplification data was only assessed up to cycle 39 for environmental samples (see methods below, and results).

To determine the initial copy number of the CO1 probe region for any given sample, a standard curve was made using a synthesized oligo (IDT GeneBlocks) of the 164 bp CO1 region. The stock geneblock oligo (1 ng/μl) was diluted to 300,000 copies based on mass, and then serially diluted down to 30 copies to complete the standard curve. The five standards (300,000 copies to 30 copies) plus a water blank (zero copies) were run in duplicate for each qPCR assay (12 standards run on each plate). Cq value, the PCR cycle at which fluorescence rises above background, was regressed against log copy number to examine the expected fit of the standards: qPCR plates (samples) which produced an r^2^ value of less than 0.98 were re-run.

### Initial testing of the assay

Initial tests of the assay were conducted using DNA samples of river herring (alewife and blueback herring) from Chesapeake Bay and Massachusetts, extracted from fin clips. Tests for assay specificity and performance were carried out using a wide range of potential non-target species that included closely related alosids (e.g. hickory shad and American shad), locally occurring fish in the family clupeidae (of which river herring are members; e.g. atlantic herring *Clupea harengus*, bay anchovy *Anchoa mitchilli*, gizzard shad *Dorosoma cepedianum*, and Atlantic menhaden *Brevoortia tyrannus*) and other co-occurring, but more distantly related fish (e.g. striped bass *Morone saxatilis* and Atlantic sturgeon *Acipenser oxyrinchus oxyrinchus*). Fin-clips were stored in 70–95% non-denatured ethanol (EtOH) prior to extraction with the Qiagen DNeasy kit (Valencia CA). The qPCR assay was run on known target (river herring) and non-target samples along with water blanks to assess amplification and assay performance in advance of testing for the presence of herring DNA in environmental water samples.

### Environmental sampling in Chesapeake Bay

Water samples were collected by wading out from shore or by boat at 196 sites across 12 tributaries in Chesapeake Bay in the spring of 2015 and 2016 (Fig 1, Table 2). Polypropylene bottles (1 L) were autoclaved before use and filled with approximately 800 mL of water from sample sites (to leave room for expansion during frozen sample storage). Care was taken to ensure that sample site water flowed directly into bottles without contamination from water contacted or stirred up by the sample collector, and that the inside of the bottle and cap were not touched. Randomly selected bottles were filled with deionized water in the lab to serve as ‘cooler blanks’. These control bottles were held in the cooler with sample bottles, and dunked in the stream (with the top closed) at a site where herring presence was expected. Water samples were transported in coolers back to the lab at Smithsonian Environmental Research Center (SERC) in Edgewater, MD, where they were frozen at -20°C until being transported to Horn Point Lab (Cambridge, MD) for filtering and extraction.

**Table 2:** Sampling sites and basic results.

Shore	River	Num. Sampling Sites	eDNA hits^1^
Eastern	Choptank	29	28/77
Eastern	Nanticoke	32	19/63
Eastern	Northeast	10	6/20
Western	Deer Creek	8	1/15
Western	Gunpowder	9	2/18
Western	James	26	11/42
Western	Mattaponi	9	2/17
Western	Pamunkey	11	4/30
Western	Patapsco	10	10/65
Western	Patuxent	16	7/32
Western	Piankatank	1	1/1
Western	Rappahannock	32	15/63
Western	York	2	0/2
**Totals**		**195**	**106/445**

^1^ number of water samples with positive hits / number of water samples taken in that tributary

Water samples for eDNA analysis were collected as part of a larger habitat use and monitoring study of river herring in Chesapeake Bay, in which parallel monitoring data was generated for river herring adult presence (and visual identification to species including hickory and American shad, if possible), and river herring ichthyoplankton abundance. Presence of adult river herring, hickory shad, and American shad was determined by visual survey of up to a 25-m section of stream, depending on site accessibility. In larger, deeper, or more turbid streams where the full water column could not be seen, or in smaller streams where river herring or shad were observed, a cast net (1.52-m radius with 9.53-mm mesh) was thrown at least three times in an attempt to capture individuals for identification to species. Visual assessment was not intended to be a comprehensive survey, but rather a rapid assessment that could be done quickly at a large number of sites. Ichthyoplankton were collected using a plankton drift net (46 cm x 30 cm with 500 μm mesh and a 200 mL cod end) following standard methods used by Maryland Department of Natural Resources (e.g. [68]). The net was deployed for 5 min, and water velocity was measured using a flow-meter (JDC Electronics Flowatch) for conversion to sample volume. Contents of the cod end were rinsed into a 500 mL Nalgene bottle using 80% EtOH, with a goal of sample preservation in ~70% EtOH. Fish eggs and larvae were sorted, identified, and enumerated under a dissecting microscope following [69, 70]. Identification of eggs to species was not possible, with results of COI DNA barcoding of individual eggs suggesting those considered to be from river herring could also be hickory shad or gizzard shad (Ogburn, unpublished data). Ichthyoplankton counts were standardized by sample volume prior to analysis. Ichthyoplankton sampling was conducted under Smithsonian Environmental Research Center Animal Care and Use Committee Study #03_01_13 and IACUC permit # D16-00392 (A3655-01). Eggs and larvae were euthanized in a solution of ~70% ethanol. All sampling procedures were approved as part of these permits and no fish were housed or experimented upon in this study.

Prior to filtering, all water samples were thawed for 4–8 hours at room temperature or overnight at 4°C and exact volumes were recorded to adjust eDNA copy estimates of positive detections (see below). Samples were filtered on autoclaved Pall filter housings using 47 mm (diameter) Whatman cellulose nitrate filters with 1.0 um pore size. This pore size and filter retains particles with macrobial eDNA produced by fish (e.g. [71]), yet allows the rapid filtration of most 1 liter samples onto a single filter disk. Filters were stored at -80°C in individual 15 ml Falcon tubes until extraction with the Omega Biotek EZNA Water kit (Norcross, GA). Filters (up to three per sample) were cut into small strips before the lysis step and were removed just prior to the first centrifugation step. Otherwise, manufacturer specifications were followed exactly. Extractions were eluted into 100 μl of elution buffer, quantified with the Invitrogen Qubit 2.0 Fluorometer (High sensitivity DNA kit), and stored at -20°C until qPCR assays were run.

Quantitative PCR was run with 4 μl of sample DNA in triplicate, along with the duplicated standards (300,000 to 30 copies), two water blanks (no template controls), and an inhibition control for each sample DNA. For the inhibition controls, an additional 300,000 copy standard was spiked with 1 μl of each unknown to quantify the positive shift (if any) in Cq value due to inhibitory compounds that may been co-purified with the DNA sample. Samples that produced Cq values below 39 (see results below for explanation of Cq cut off) in at least two of the three reactions were considered positive eDNA detections (river herring present) and relative abundance (in mtDNA copy number) was calculated and reported for each detection. Gene copy number estimates were adjusted for each sample (*i*) based on the volume of water filtered (standardized to 1 Liter):

$${adjusted\;copy}_{i}=[1000ml÷{sample\;vol}_{i}\;(ml)]*{eDNA\;copy\;number}_{i}.$$

Because 4 μl of DNA (out of 100 μl eluted) were run in each qPCR reaction (in triplicate), copy number reported (eDNA copies) represents copies per 40 mL of water sampled.

### Validation of qPCR results and species identification via Sanger sequencing

Each eDNA sample that produced a positive qPCR detection (at least 2/3 Cq values lower than 39) was tested for species identity (one of the two river herring species expected) via Sanger sequencing in re-amplifications of the DNA sample without the probe (e.g. using the forward and reverse primers only). PCR reactions were performed on the Biorad CFX96 with the 2x BioRad SYBR Green mastermix, and amplicons were sequenced on an ABI-3730 capillary sequencer in the forward direction at the Arizona State University DNA lab. Resultant sequences were then examined by eye with Chromas Pro (v.2.15; Technelysium Pty), compared with known, reference sequences, and confirmed with BLAST searches to the entire NCBI ‘nt’ database. For sequences that showed multiple peaks at a diagnostic SNP within the amplicon (amplicon bp 104: T/C, alewife/blueback herring) we used QSVAnalyzer v. 6-12-2012 [72] to quantify the ratio of the peak heights and infer relative proportion of species DNA from the raw sequence files. The QSVAnalyzer software examines the heights of the peaks from the preceding and succeeding 6–10 bases around the SNP and calculates an adjusted peak height ratio at the focal SNP that corrects for base-specific variation in peak height. We demonstrated the accuracy of inferring species DNA proportions from nucleotide peak heights with tests of *in vitro* mixes of fin-clip extracted DNA from the two river herring species at a range of known ratios (e.g. 1:1, 1:2, 1:4, 1:10 alewife:blueback and blueback:alewife DNA), which were PCR amplified, sequenced, and run through QSVanalyzer. Known proportions of species DNA (proportion of alewife DNA used) were regressed against DNA proportions inferred from peak height analyses, using both corrected and uncorrected approaches, to examine correspondence and accuracy of this approach before applying it to environmental samples.

### Spatio-temporal mapping of eDNA and correlation analysis

Presence or absence and relative abundance of river herring based on the qPCR eDNA data were characterized across Chesapeake Bay to examine gross spatial patterns of detection and habitat use during spring of 2015 and 2016. Environmental DNA abundance data (mtDNA copy number) and PCR inhibition data were mapped spatially across Chesapeake Bay sampling locations. Temporal patterns of eDNA abundance data over the spring months were also examined. Finally, patterns of eDNA abundance were examined across the two shores of Chesapeake Bay (eastern and western) with the Susquehanna River the dividing line between the two (Fig 1). Spatial and temporal analyses were conducted for river herring as a species group, and for each species separately, based on the BLAST results of Sanger sequences produced for each eDNA detection, apportioning copy number (abundance) between the two species based on the relative peak height ratios produced by QSVAnalyser (see above). Variation in the proportion of samples with inhibition across rivers was assessed using the Marascuilo procedure [73], which permits simultaneous testing of the differences in proportions among all pairs of rivers. In order to examine the correspondence of eDNA data to traditional methods of abundance estimation (i.e. visual observations of adults and ichthyoplankton counts at these sites), non-parametric correlation (Spearman’s Rho) was performed on the quantitative data (ichthyoplankton count vs. eDNA copy number), and Pearson’s Phi-Coefficients were calculated to assess correlation between presence/absence (detection or no detection) data for adult vs eDNA data sets and ichthyoplankton vs eDNA data sets. All statistical analyses were performed in R v. 3.4.3 [74] with maps and figures produced using the ‘ggplot2’ and ‘ggmap’ packages [75, 76]. River herring abundance data (eDNA, adult, ichthyoplankton) including location and date information, are deposited in Dryad (doi:10.5061/dryad.n60t8b3).

## Results

### qPCR assay results and performance

**Fig 2:**
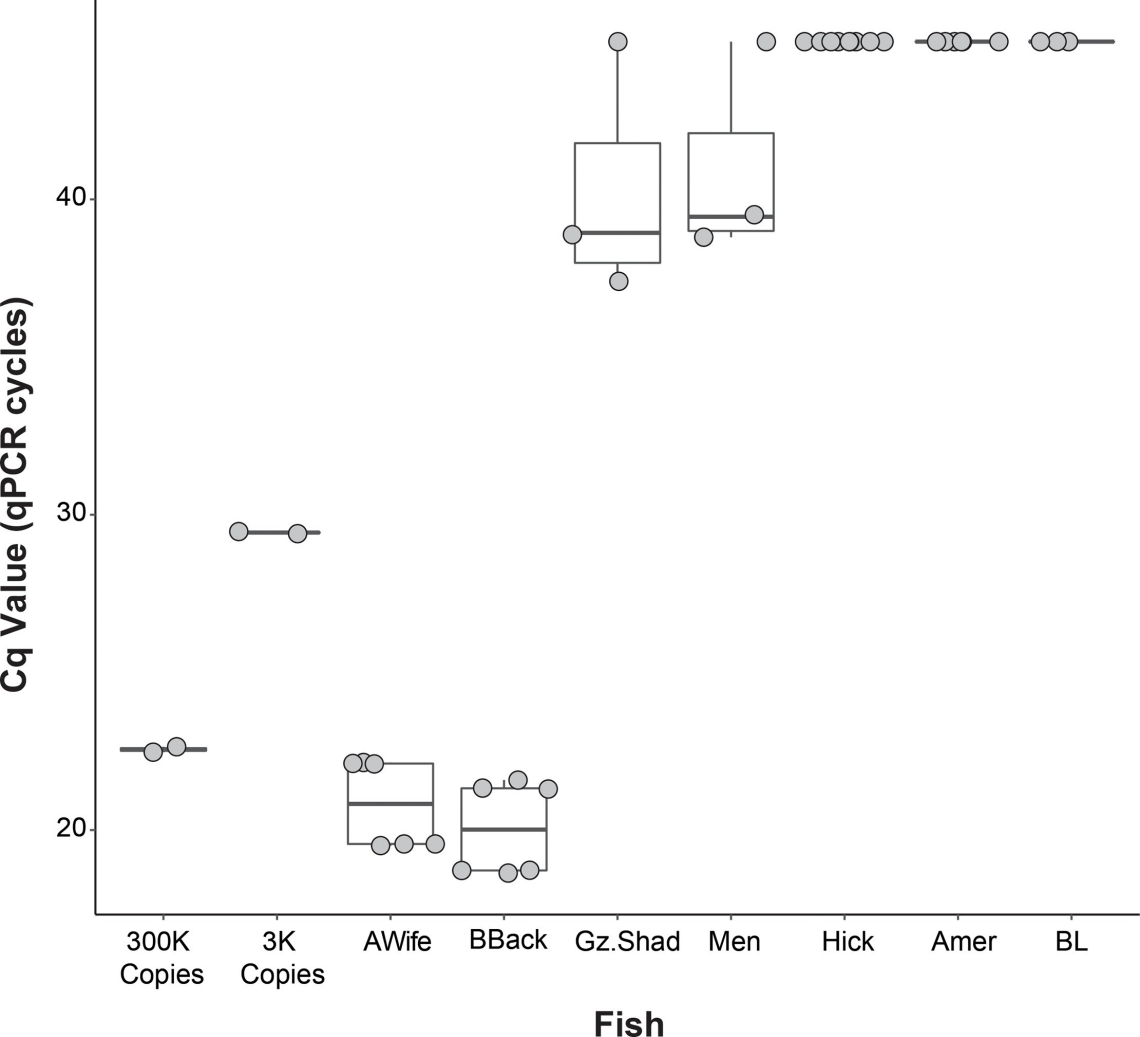
qPCR results for lab trials of target and non-target fish. 300K and 3K copy samples are the PCR results for respective synthesized standards on the standard curve, ‘GzShad’ is gizzard shad, ‘Men’ is menhaden, ‘Hick’ is hickory shad, ‘Amer’ is American shad, and ‘Awife’ and ‘BBack’ are alewife and blueback herring, respectively. BL is a no template control (blank).

Tests of the molecular beacon assay with fin-clip extracted DNA from river herring and a number of non-target fishes showed that the assay was highly specific to river herring (Fig 2). No amplification (no detectable increase in fluorescence after 45 qPCR cycles) was ever observed for hickory or American shad, which are the two most closely related alosids to river herring and often co-occur with river herring within river systems in Chesapeake Bay [77]. Most other non-target species also lacked amplification completely; however, inconsistent, weak (late) amplification was detected for DNA samples from gizzard shad and menhaden, with one of three samples producing a Cq value as low as 38–39, though other replicates did not amplify (>45 cycles). This occurred in lab trial qPCR reactions with 50 ng of purified, fin-clip DNA, and no amplification was observed with less DNA (20–25 ng). In contrast, amplification of river herring fin clip DNA was robust, consistent, and much stronger, producing Cq values of ~18–24, which is similar to the Cq values produced by the 300,000 copy standard of the synthesized oligo for the 164bp CO1 region (Fig 2). To be conservative, only Cq values 39 or below were considered as true eDNA detections in downstream analyses of environmental samples (see below). Sanger sequencing of trial qPCR amplifications of river herring DNA confirmed that the expected 164 bp region was amplified from river herring (alewife or blueback herring).

To explore the potential for estimating relative abundance of alewife or blueback herring DNA in mixed environmental samples, we examined peak heights at a diagnostic nucleotide (bp 104: alewife-T, blueback-C) in sequences generated from of a series of ‘mock’, known- concentration mixes of river herring DNA pools. Sequencing of these mixed DNA pools revealed a very strong correlation between expected DNA ratios and QSVAnalyzer estimates of species proportions from peak heights over a range of DNA ratios (1:1 to 1:10). As shown in S1 Fig, there was high correspondence between the expected proportion of each species DNA using both the corrected (linear regression P = 1e-10, R^2^ = 0.968) and uncorrected methods (linear regression P = 1e-12, R^2^ = 0.967). Correcting peak heights via proximal base height averages around the variant site appeared to improve the accuracy of the species composition estimates slightly, with a slope that was closer to the expected 1. Corrected peak height ratios were used to estimate the number of eDNA copies from each species in positive detections with both alewife and blueback herring DNA.

### Environmental sampling and eDNA abundance across Chesapeake Bay

**Fig 3:**
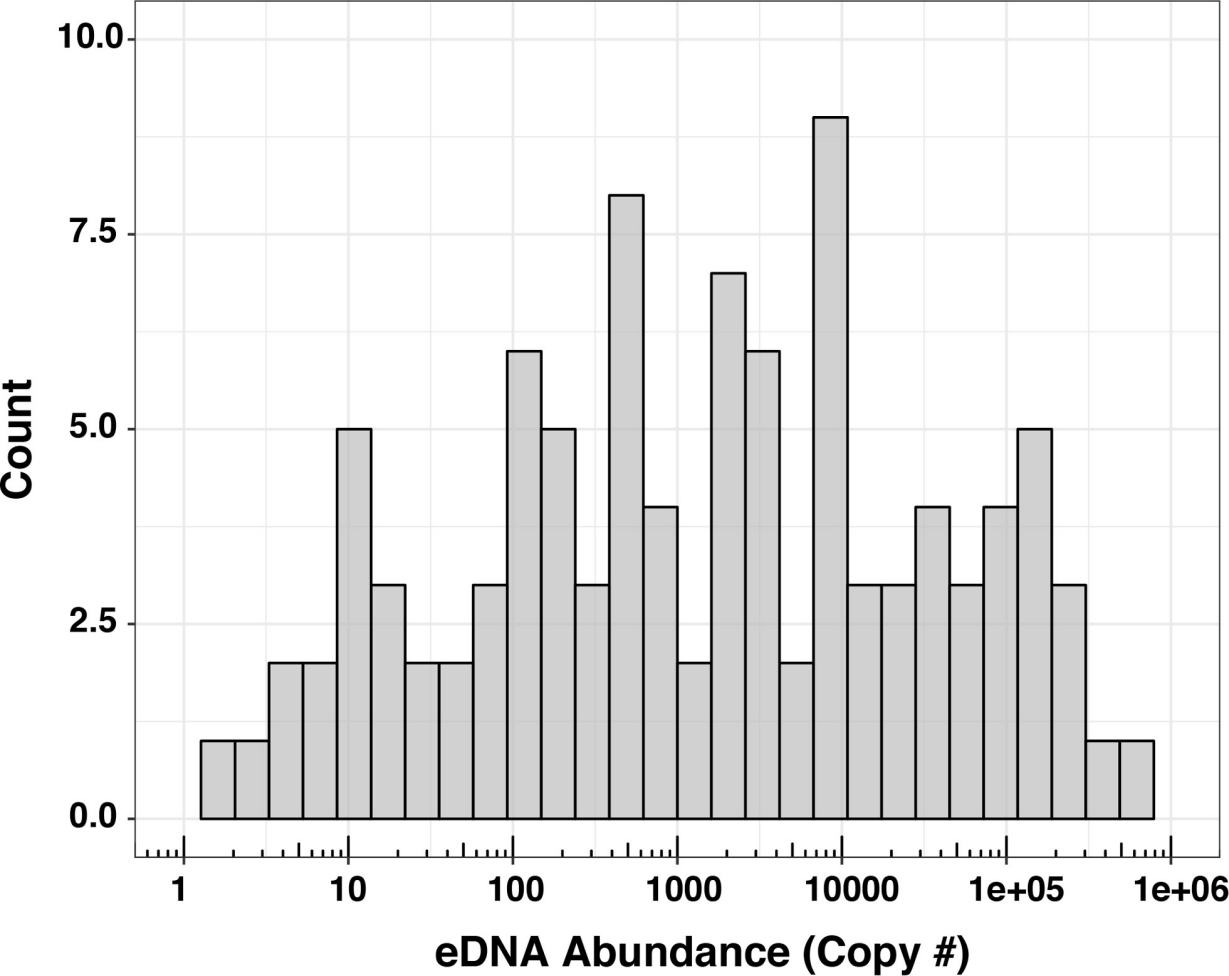
Histogram of positive eDNA detection copy numbers from 2015–2016. eDNA abundance (copy number) is plotted on a log scale from 1 copy up to 1 million (x-axis), with the count of occurrences (number of samples) within each histogram bin on the y-axis. eDNA copy numbers for each sample reflect the initial number of mtDNA copies per 40 mL of water filtered.

**Fig 4:**
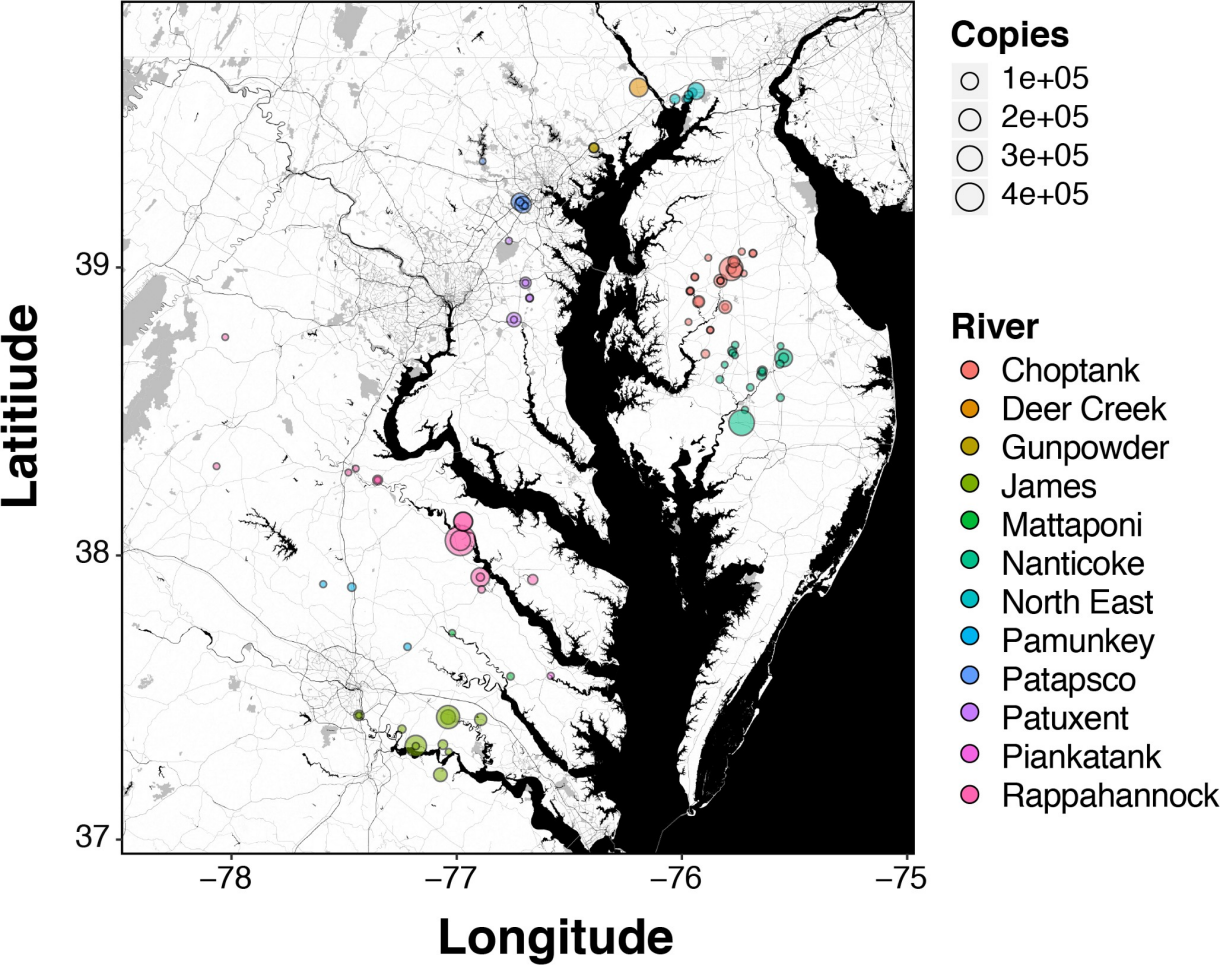
Map of positive river herring eDNA detections across Chesapeake Bay. Size of data points (positive detections) proportional to the magnitude of amplification (mean mtDNA copies) and are colored by tributary. eDNA copy numbers for each sample reflect the initial number of mtDNA copies per 40 mL water filtered.

Between 2015 and 2016, 462 1-liter water samples were collected from a variety of locations within river systems on the eastern and western shores of Chesapeake Bay (Fig 1). An additional 45 water samples were collected as field or “cooler” blanks (deionized water samples brought to the field and dunked in the sampling stream) that were blind to molecular lab technicians (sent with normal river/site labels), to test for lab, handling, or transport-based contamination. None of the 45 controls produced any amplification. Of the 462 environmental samples taken, 445 were processed in the lab for eDNA analysis, 23.82% of which generated positive eDNA detections (106/445, Table 2). The magnitude of eDNA abundance (starting copy number) ranged from ~ 1 copy up to 499,066 copies, with a mean of 31,388 copies and median of 1,771 copies; most (78%) positive detections were under 100,000 copies (Fig 3). The number of positive eDNA detections was fairly consistent between years (46 out of 201 samples analyzed in 2015, 60/244 in 2016; Fisher test P = 0.73) despite sampling of different river systems across years. River herring eDNA was detected across most of the major river systems, as shown in Fig 4, with multiple, large magnitude (high copy number) detections in the Northeast, Choptank, and Nanticoke rivers on the eastern shore, and the Patapsco, Rappahannock, and James rivers on the western shore. PCR inhibition, quantified as a three cycle or greater positive shift in Cq values of the spiked control, was detected in 38.3% of the samples overall, and differences in the proportion of samples showing inhibition were detected across rivers (Chi-square homogeneity test, P = 7.5e-06; S2 Fig). Inhibition appeared to be higher in slower-moving, coastal plain streams that have more dissolved organic matter (e.g. Choptank River, James River, Nanticoke River, Rappahannock River) compared with faster moving, piedmont streams (e.g. Deer Creek, Northeast River, Gunpowder River, Patuxent River). Using the Marascuilo approach for pairwise comparisons of inhibition proportion among rivers [73] significant differences were observed for eight river comparisons, all of which were between a piedmont steam/river (Gunpowder, Northeast, or Deer Creek) and a coastal plain stream/river (Nanticoke, James, Choptank, Mattaponi—tributary of the York River).

**Fig 5:**
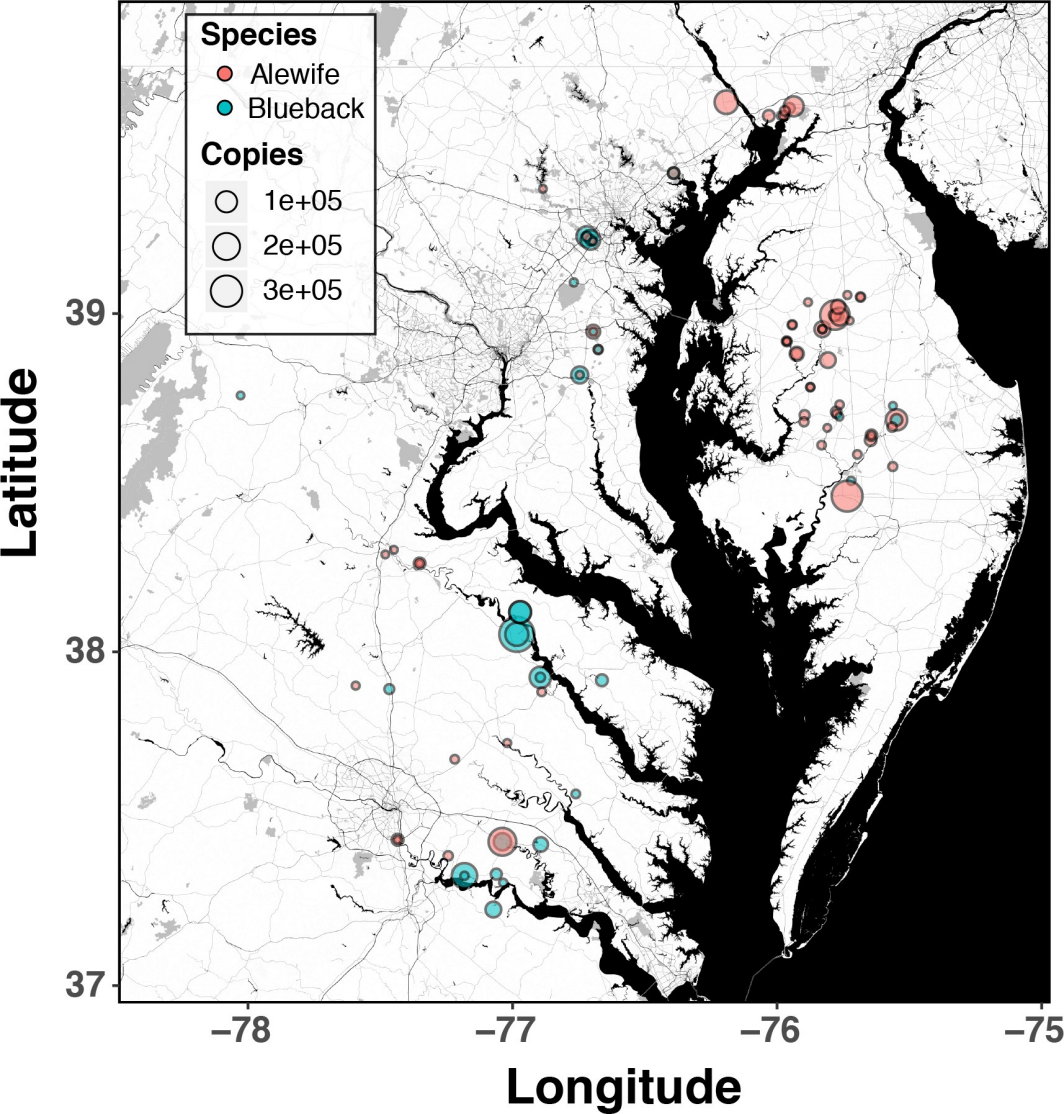
Map of positive eDNA detections broken down by species (alewife or blueback herring). Size of the point is proportional to the magnitude of amplification (mean mtDNA copies). eDNA copy numbers for each sample reflect the initial number of mtDNA copies per 40 mL of water filtered.

**Fig 6:**
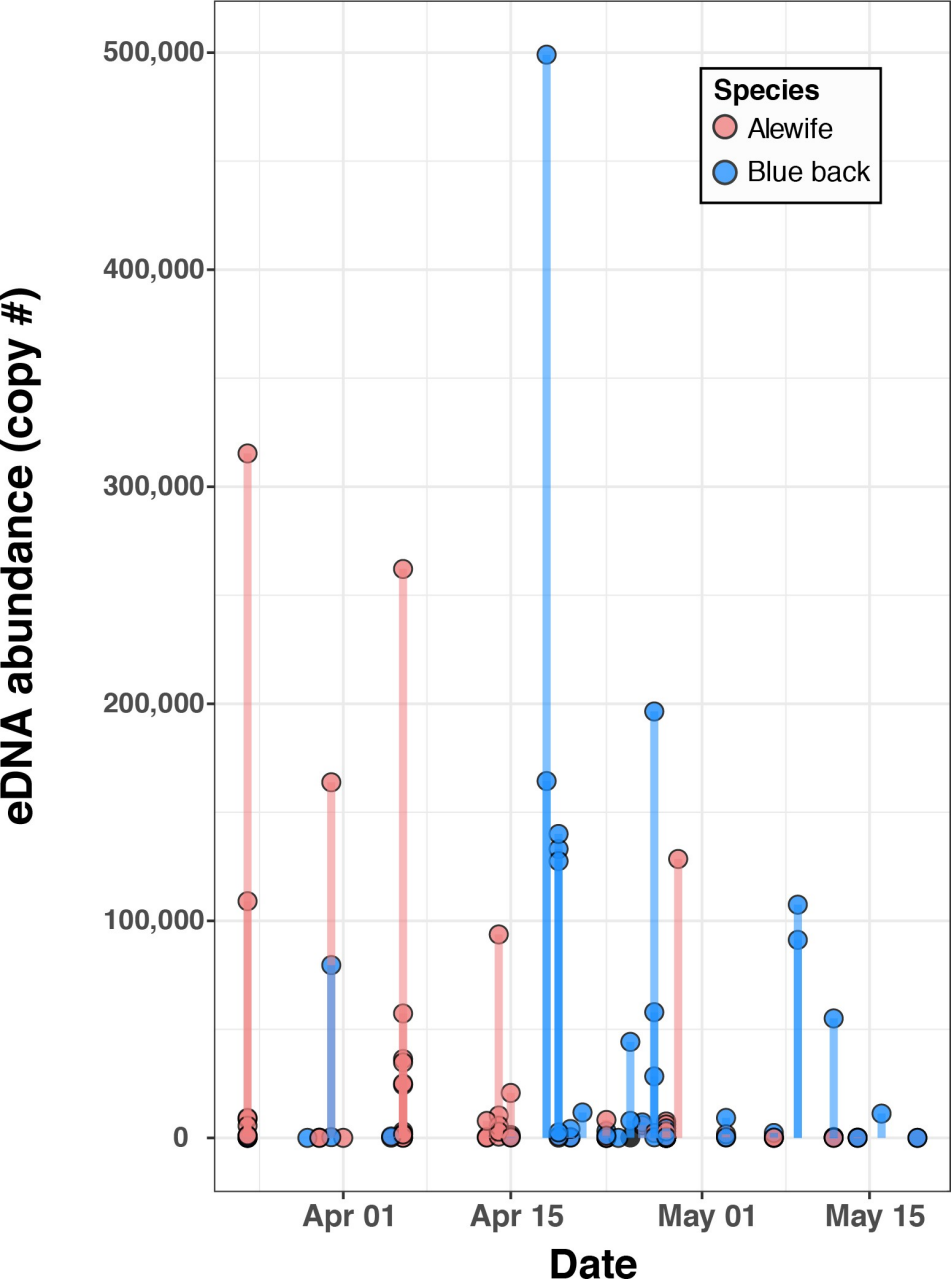
Temporal distribution of eDNA copy number (abundance) by species, during spawning. Data for years 2015 and 2016 combined. Multiple data points (filled circles) at the same date (i.e. same x-axis coordinate) reflect samples taken either on the same day at different sites (same year) or the same date across years.

Post-qPCR validation of positive hits (Cq values under 39) with Sanger sequencing produced readable sequence data for 104/106 samples, with 102/104 showing 100% identity (NCBI Blast-n; E-values <1e-35) to alewife (N = 53), blueback herring (N = 30) or both (multiple peaks at diagnostic C/T SNP at 104bp; N = 19). Sequences from two samples (no polymorphism at 104bp) matched blueback herring at 95–97% identity, and local BLAST searches to SERC sequences used as part of our assay development revealed 100% match to hickory shad, which did not have a CO1 sequence published in NCBI Genbank at the time of the analysis. Using the corrected peak height ratios at the diagnostic C/T SNP permitted calculation of the number of mtDNA (CO1) copies for each species in multiple-species samples, by multiplying the relative ratio of the T:C or C:T peak heights by the eDNA copy number for alewife and blueback herring, respectively. Including these additional data points, spatial and temporal comparisons of species-specific patterns of eDNA detections in 2015 and 2016 revealed that both alewife and blueback herring are found across most rivers, as expected, but with very different detection probabilities on the eastern and western shores of Chesapeake Bay (Fig 5: see S5 Fig for breakdown by year). On the eastern shore, 46 vs. 12 positive eDNA hits were identified as alewife and blueback herring respectively, while on the western shore, 26 vs. 37 hits were identified as alewife and blueback herring respectively, the ratios of which are significantly different than expected by chance (Fisher exact test P = 2.09e-05). In general, eDNA detection probability was much higher on the eastern vs. western shores (53 out of 160 samples with positive detections on the eastern shore vs. 52 out of 284 samples with positive detections on the western shore; Fisher exact test P < 2.0e-12). Finally, variation in species-specific eDNA relative abundance (magnitude) and detection probability showed an interesting temporal pattern, with stronger and more frequent eDNA detections earlier in the season for alewife (March—April 15; Mann-Whitney U test, p = 0.004), versus stronger, more frequent detections for blueback herring later in the spring (April 15 –mid May; Mann Whitney U test, p <0.0001; Fig 6). This result follows reported differences in the spawning biology of the two species, in which alewife migrate and spawn at lower temperatures (i.e. earlier in the spring) than blueback herring [78–80, 27].

### Correlation between eDNA and traditional methods of abundance estimation

**Fig 7:**
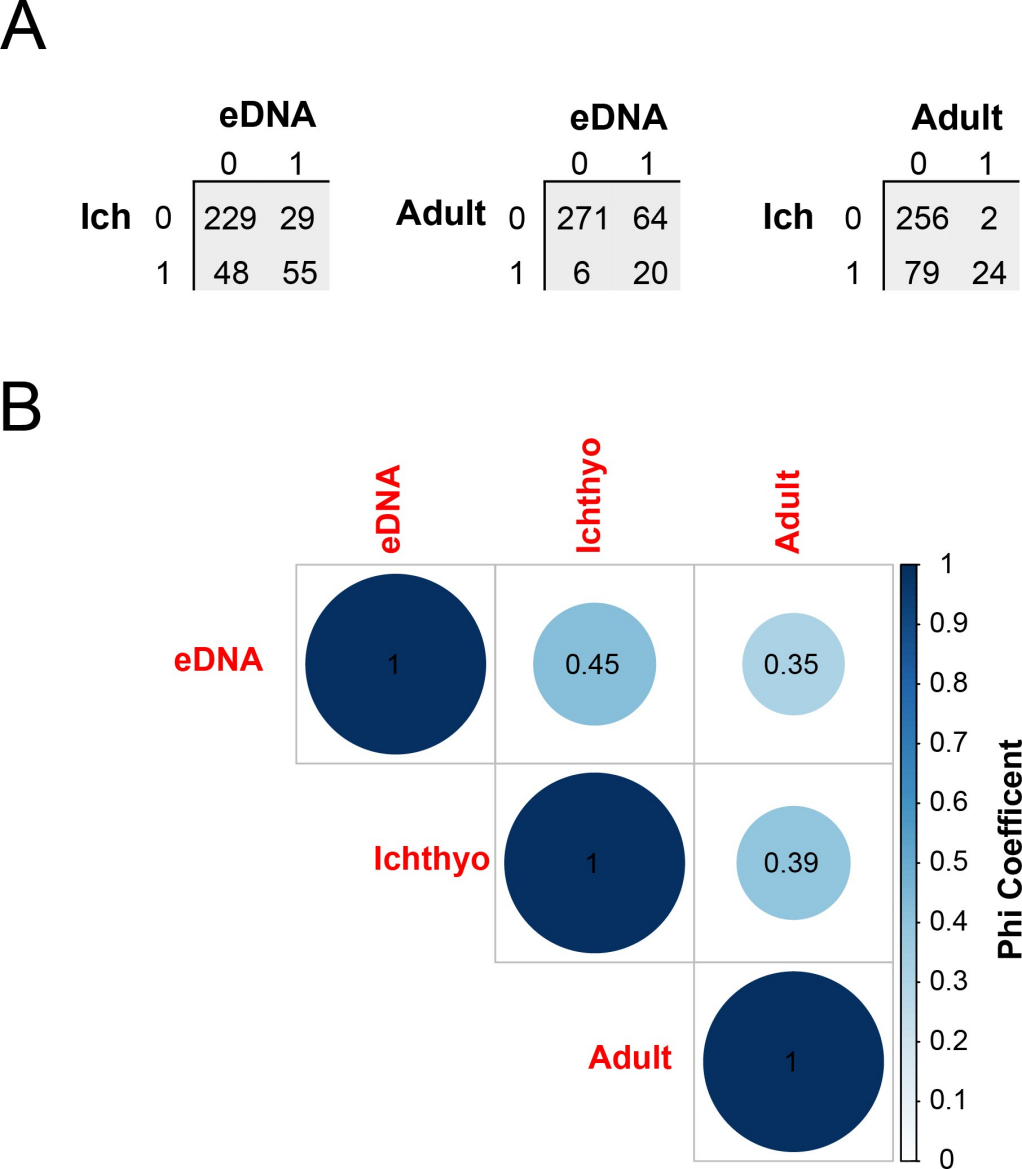
Correlation results (Phi coefficients) among the abundance metrics. Panel A shows the summary tables for each pairwise comparison (0 = no detection, 1 = positive detection); panel B shows a multiple correlation plot displaying Phi-coefficient values, with filled circles proportional to the magnitude of the Phi coefficient.

While the eDNA data provide interesting patterns of detection probabilities and changes in river herring abundance over space and time, it is also important to consider how they compare to data from traditional abundance estimation approaches such as ichthyoplankton sampling and adult observations. Data for 361 sampling events were available across all three data sets (i.e. no missing data on a given date/location for ichthyoplankton, adult, or eDNA data sets), and were used for subsequent correlation analyses. First, we examined correlation between data sets based on presence/absence data (i.e. eDNA hit/observation = 1 or no eDNA hit/observation = 0), which allowed inclusion of the adult observation data set. Summary cross-tables and pairwise Phi-coefficient estimates are shown in Fig 7. We found statistically significant correlations among all three measures (P < 5.0e-12), with highest Phi-coefficient estimates between eDNA and ichthyoplankton (0.45) and lowest Phi estimates between adult and eDNA data sets (0.35). We also examined the association between the quantitative eDNA abundance data (number of mtDNA copies in 1 L of water from a given sample) and ichthyoplankton count data, the sum of all eggs and larvae identified as river herring in a given sample, using Spearman’s-Rho statistic, a non-parametric correlation test appropriate for zero-inflated data. The Spearman correlation coefficient was high and very significant (Spearman’s Rho = 0.52, P < 2.2e-16). Moreover, distribution-free permutation tests also confirmed that a value this high was extremely unlikely to occur by chance (S3 Fig). Regression of log (base 10) eDNA copy number on log (base 10) ichthyoplankton counts also showed significant dependence or correspondence between the two measures (R^2^ = 0.40, p = 2e-16; S4 Fig). There is significant spread around the regression line, but there appears to be a very strong relationship between these two estimates of river herring abundance.

## Discussion

Recent declines in river herring abundances across their US east coast range have prompted more intensive monitoring efforts and spurred the development of alternative approaches to assess run strength and characterize patterns of river herring habitat use across human-mediated landscapes. Environmental DNA (eDNA) sampling represents a promising alternative approach to detect and quantify river herring abundance in aquatic environments that is highly sensitive but less invasive and potentially less labor-intensive than traditional capture-based survey approaches. In this study, we developed and tested a qPCR assay to detect the presence and relative abundance of river herring in tributaries of Chesapeake Bay, with the goal of providing an additional tool for river herring monitoring. Overall, the eDNA assay showed very high target specificity, performed well across a range of field samples and environments in Chesapeake Bay, recovered interesting spatial and temporal patterns among the species, and produced abundance (copy-number) and presence/absence data that corresponded well with visual surveys of adults and net counts of larvae and eggs (ichthyoplankton sampling).

### Assay specificity and performance

Lab trials with DNA extracted from a variety of ecologically relevant (co-occurring) or phylogenetically similar species (e.g. alosids and clupeids) demonstrated that the herring qPCR assay had very high target specificity (Fig 2). Observations of late and inconsistent amplification in menhaden and gizzard shad (38 or 39 cycles and above) were possibly due to fewer mismatches of the probe for these species (e.g. at the probe site, menhaden sequences had one less nucleotide mismatch compared with hickory shad sequence). The design of the molecular beacon probe was focused primarily on excluding closely related alosids (American and hickory shad), which presented a significant challenge in assay design due to the high similarity in CO1 sequence (e.g. only ~3–5% divergence over 650 bp of CO1 between river herring and hickory shad). Amplification of off-target species was only seen for high-concentration fin-clip DNA templates, and only in the later cycles of the qPCR run (>38 cycles). Moreover, Sanger sequence validation of positive eDNA detections from the field showed that PCR amplicons derived from river herring DNA in all but two cases, in which the sequence was identified as hickory shad. However, it is important to note that the Sanger sequence validation used only the forward and reverse primers (no probe), and thus was much less specific than the full probe set, which never amplified hickory shad in lab trials; Fig 2). Overall, the molecular beacon assay developed here proved to be highly specific to river herring, and produced reliable, repeatable eDNA abundance data in lab and field trials.

One of the drawbacks of our single probe qPCR design was that it could not distinguish between the two river herring species in a single assay reaction, requiring subsequent sequence validation of each positive eDNA detection to identify which species was present. Though the sequence validation provided a useful quality check on each positive detection, it added some additional labor and cost. Our initial attempts to develop a dual-probe assay (one for each river herring species) were thwarted by the relatively few sequence differences between the two river herring species (~8 individual base differences across 650bp CO1, ~98.8% similar). In fact, there are three diagnostic SNPs across the 164 bp amplicon of our probe assay, which clearly allows discrimination of blueback herring from alewife based on full, Sanger sequence data, but we were unable to design a suitable qPCR primer/probe set around these particular nucleotides that also distinguished (lacked complementarity to) hickory and American shad. A high-throughput metabarcoding approach targeting the CO1 region (e.g. [40, 81]) or other gene regions would likely solve this, or the design of a qPCR assay for a nuclear marker might provide better resolution of the two species and facilitate the development of a dual-probe assay for species discrimination in a single reaction. For now, however, this qPCR assay appears to be very robust and reliable, and is deployable on a scalable, as-needed basis with variable throughput (1 or 500 samples), using technology and expertise that should be available at most molecular labs with a quantitative PCR machine.

Finally, it is important to note that PCR inhibition was observed in a number of field samples, the frequency of which appeared to vary by river, with coastal plain streams exhibiting more frequent and sometimes complete PCR inhibition compared to piedmont streams (S2 Fig), which we speculate could be due to elevated organic matter content. Though the DNA extraction kit we used is designed for isolating DNA from water samples and includes reagents that facilitate the removal of potential inhibitors such as humics, going forward, additional DNA cleanup steps will need to be incorporated into the eDNA pipeline.

### Comparison of eDNA detection to traditional catch methods

Correlation and regression analyses using multiple approaches (parametric, non-parametric, and permutation-based) indicated that relative abundance of river herring estimated via eDNA copy number from field samples corresponded very well to traditional abundance estimation methods. Pairwise Phi-coefficients calculated among all three datasets for presence/absence data, revealed higher correlations between eDNA and ichthyoplankton compared with eDNA and adult observation data (Phi = 0.45 vs 0.35, for eDNA-Ichthyoplankton and eDNA-Adult data, respectively; P<0.0001 for both comparisons). Adult presence data were generally more sporadic in their collection due to variable flow conditions, site access issues (dense vegetation in late spring), or user conflicts (we were not permitted to cast net in Maryland at sites with recreational anglers present) that prevented or complicated observation at certain sampling sites on particular days. Because our adult sampling methods were designed for rapid assessment rather than comprehensive sampling (e.g. [82]), we expected that the eDNA assay would detect river herring where adults were present, but would also detect them in many places where adults may have been present but were not observed in the adult survey. Sampling adults can be particularly challenging due to the episodic nature of the spawning run [27]. Ichthyoplankton surveys are likely to be more sensitive as they capture any plankton or eggs traveling downstream over a certain period of time and don’t require good visibility in the water, though identification of eggs and even small larvae to species by observation can be difficult. Correlation analysis using quantitative abundance data revealed very good association between ichthyoplankton and eDNA data sets (Spearman’s Rho = 0.52), further solidifying the strong relationship between eDNA and early life-stage abundance data.

The finding of strong correlations between river herring adult and ichthyoplankton observations and eDNA abundance adds to a small but growing number of studies that have investigated the relationship between animal density metrics and eDNA abundance in field samples across a range of species. For amphibians, [33, 37, 38] showed significant correlations between biomass or density and eDNA. For fish, [36] showed significant correlations between biomass and eDNA in experimental ponds and [35] found that probability of detection increased with relative abundance of fish species. More recently, [31] reported that eDNA abundance of lake trout correlated well with gill net catch per unit effort data and [83] reported high correlations between biomass/density and eDNA abundance of a freshwater fish. Clearly, estimates of environmental DNA abundance (e.g. copy number or DNA concentration) can produce accurate information about animal densities or animal presence/absence that correlate well with estimates from traditional, capture-based approaches.

Despite highly significant correlations between river herring eDNA abundance and ichthyoplankton counts, the fit of the regression of these variables was rather poor (R^2^ = 0.40; S4 Fig) compared to previously published data from freshwater systems or pond/mesocosm studies (e.g. [83, 36, 37]). This result might indicate that the predictive power of eDNA to estimate river herring abundance in these rivers may be somewhat limited; however, there are a few factors related to our sampling approach or analyses that might be obscuring this relationship. First, ichthyoplankton collected in nets are often not identifiable to species (especially not eggs), and thus, positive counts may include species other than river herring. A number of the ichthyoplankton ‘positive’, eDNA ‘negative’ (no detection) data points could actually be true negatives because eggs/larvae collected were from other, morphologically similar species (e.g. hickory shad). Second, eDNA abundance estimates may be decoupled from ichthyoplankton counts if detections are based on eDNA from adults that are present in the river system. Adults may enter the system before they spawn or may reside for some time after spawning, so positive eDNA detections of adult eDNA may appear to conflict with negative ichthyoplankton counts when in fact they are representing true adult eDNA detections. Finally, variation in environmental conditions across these rivers (e.g. flow, organic matter, salinity) may also have contributed to variable eDNA detection. For example, we observed clear differences in PCR inhibition across rivers and among stream types (piedmont vs. coastal plain; S2 Fig) that may be related to variation in organic matter content. If samples with evidence of inhibition (i.e. spiked standard Cq value increased by 3 or more) are removed from the analysis, the Phi-coefficient estimate of correlation between eDNA and ichthyoplankton data actually increases (from 0.45 to 0.52), as does the Spearmans correlation (0.52 to 0.60) and the log-log regression r-squared (from 0.40–0.48), indicating that PCR inhibition has had at least some effect on our analyses. Variable flow conditions also made ichthyoplankton sampling difficult in certain instances and variable transport speeds or transport distances of eDNA may alter detection probabilities substantially (e.g. [46, 49]). Few previous studies have examined eDNA abundance across a large spatial range and in such a dynamic tidal river system like Chesapeake Bay, so it is not clear how comparable these data are to those from previous studies. Future efforts to refine inferences of animal density or biomass from eDNA concentration will, of course, require more sophisticated incorporation of environmental data (e.g. temperature, flow; [46]) into statistical models of detection probability that can adequately address some of the inherent issues of eDNA sampling (e.g. over-dispersion; [42]).

Given the timing of eDNA sampling during river herring spawning in the spring, the higher correlation between eDNA and ichthyoplankton data might indicate that the eDNA signal is coming primarily from gamete, embryo or larval DNA. Gametes are the product of spawning adults in the river system, so the relatively lower correlation between eDNA and adult data could simply reflect the reduced quality or prevalence of adult observation data. An important shortcoming of eDNA sampling is that it is difficult or impossible to know the life stage source of the eDNA, and in our study, it is impossible to know the relative contribution of eDNA sources (adult or offspring) to the eDNA data collected. Nevertheless, eDNA sampling during spawning may be more effective in general (higher detection probability) due to the increased amount of gamete-derived eDNA or adult-derived eDNA (sloughed cells, feces, mucous) during spawning over a geographically restricted area. Indeed, other studies have shown that eDNA detection is likely influenced by seasonal activity or season-specific behavior of salamander species (e.g. [84]). Sampling of rivers after river herring spawning is complete, as adults are exiting a particular river system, and comparing detection probabilities to those during spawning, may shed light on the relative difference in detection probabilities between adult vs. gamete eDNA in the environment.

Finally, the temporal pattern of species-specific abundances revealed by the eDNA data further demonstrates the ecological relevance of the assay. Alewife eDNA detection probability and relative abundance was higher in rivers earlier in the spring, when water temperatures were lower (earlier spawning or migration), while blueback herring eDNA was detected with greater frequency and magnitude later in the spring (later spawning or migration; Fig 6). This is a significant result because it demonstrates that the eDNA data alone can recover differences in species biology (migration or spawning timing) that have been widely observed and reported in the literature (e.g. [78–80, 85, 27]). Moreover, from a practical standpoint, this finding suggests that eDNA may be a useful tool for tracking the species-specific patterns of spawning timing or initiation of migrations up river, which has significant potential management use. Overall, based on the correlation results and the observed temporal patterns of eDNA abundance data that align with known differences in species biology, it is clear that eDNA-based detection of river herring provides high quality data on presence and relative abundance that corresponds well to more traditional estimates of presence and density.

### Spatial patterns of eDNA detection and abundance

In general, river herring were detected fairly equally across years and across most tributaries in Chesapeake Bay, but a couple of interesting spatial patterns emerged. First, we found that eDNA detection probabilities were much more likely on the eastern (33% samples with positive eDNA detection) vs. western (18% samples with positive eDNA detection) shores. A similar disparity in detection probability was also observed for the ichthyoplankton data (46% and 17% detection probability of ichthyoplankton on the eastern vs. western shores, respectively; Fisher exact test P< 2.0e-08), indicating that this was not an artifact of the eDNA method through PCR inhibition or some other mechanism. At face value, these findings would suggest that river herring may be more abundant on the eastern shore; however, we stress that this is only a preliminary observation and more targeted and repeated sampling is needed to confirm this result. The potential explanations for the observed disparity in river herring detection between shores are numerous, but one obvious possibility is that habitat or land use might drive the abundance differences. For example, the eastern shore of Maryland is notably more rural with a greater proportion of land use dedicated to agriculture, while the western shore is significantly more developed and urbanized, with more impervious surfaces and reduced riparian buffers, which can have important consequences for stream ecosystems (e.g. [86]). Thus, it is possible that rivers in less developed watersheds can support greater abundances of river herring. However, it could also be argued that watersheds that are heavily influenced by agriculture production could have reduced water and habitat quality due to fertilizer runoff and nutrient loading, balancing out the advantages of reduced development. It is also possible that the eastern shore has historically (i.e. from pre-industrial times) supported more river herring due to any number of physical, biological, or habitat features of the particular rivers that we sampled, which may be completely unrelated to recent human activities. Genetic differences between eastern and western shore stocks of both alewife and blueback herring have also been observed using microsatellite markers [87], adding additional uncertainty in the potential causes of patterns in abundance.

Another interesting spatial pattern that emerged was that the probability of detecting the two river herring species differed significantly between the shores of Chesapeake Bay, with alewife more common among eastern shore eDNA detections and blueback herring more common among western shore detections. This result follows the above-mentioned finding of relatively lower detection probabilities for river herring on the western shore overall (where blueback herring are more likely, but detected at lower frequency). Of course, it is not clear whether land use/habitat features, inherent biological or physical characteristics of rivers that pre-date major anthropogenic forces, genetic structure, or a combination of these factors are driving differences in species-specific occupancy rates within rivers or across shores. Again, we can only speculate about the potential causes of the disparate detection probabilities of the two species across Chesapeake Bay shores, but this is a very interesting result that, to our knowledge, has not been documented in previous surveys of river herring abundances in Chesapeake Bay tributaries. A much more comprehensive and sophisticated modeling of habitat use by river herring that integrates multiple forms of abundance data (including eDNA) and a full analysis of spatial GIS/landscape variables is required to more completely understand spatial patterns of river herring abundance and distribution, and is the subject of ongoing work. However, this preliminary analysis of spatial differences in eDNA detection probabilities demonstrates the potential power of eDNA sampling for rapid assessment of the distribution and abundance of river herring or other fish species across broad spatial scales.

## Conclusions

Overall, this study demonstrated that our development of a qPCR assay to detect river herring eDNA in estuarine and freshwater habitats was successful, discriminating the target species in both lab and field samples. Sampling 196 sites among 12 tributaries of the Chesapeake Bay revealed interesting and plausible spatio-temporal patterns of eDNA detection that corresponded well with traditional methods of abundance estimation. Environmental DNA surveillance holds great promise as a complementary tool to monitor river herring, which have seen acute recent declines in the US Mid-Atlantic. This study adds to the growing literature using eDNA to detect the abundance of fish in the environment, and results suggest, again, that abundance or detection information derived from eDNA data has high correspondence with traditional catch or observation-based data.

## Supporting information

S1 File CO1 FASTA format alignment. (FASTA)

S1 Fig Comparison of expected alewife/blueback DNA ratios to observed peak height ratios. Plot of expected species DNA ratios (from 1:10 to 10:1 alewife:blueback DNA added to PCR) vs. observed DNA ratios inferred from peak height ratio analysis using QSVAnalyser at the diagnostic SNP at bp 104 (alewife = T, blueback = C) after sequencing the PCR amplicon. Ordinary least squares (OLS) regression lines are plotted for raw (red) and corrected (blue) peak height ratios compared to a 1:1 line (dashed) alongside OLS equations and R^2^ values (below the curves for the raw peak height ratios, above the curves for the corrected peak height ratios).
(TIF)

S2 Fig Box and whisker plots of PCR Inhibition in environmental samples across rivers. For each river or steam (grouped by type: piedmont, coastal, and mixed) inhibition is plotted as the number of additional qPCR cycles (Cq values) of the 300,000 copy oligo standard after spiking it with a given environmental sample. Note the break in the Y axis–samples that produced no amplification (complete inhibition) are plotted at ‘No Amp’.
(TIF)

S3 Fig Histogram of permuted Spearman’s correlation estimates. Histogram of Spearman’s Rho values from 1000 random permutations of the eDNA and ichthyoplankton datasets. The dashed red line shows the observed estimate of correlation between eDNA and Ichthyoplankton abundance datasets (Rho = 0.52).
(TIF)

S4 Fig Plot of eDNA abundance vs. ichthyoplankton count. Data are plotted on a log-log scale. eDNA copy numbers reflect the initial number of mtDNA copies per 40 mL of water filtered.
(TIF)

S5 Fig Maps of river herring eDNA detections across two years (spring 2015 and 2016) in Chesapeake Bay, USA. Size of data points (positive detections) are proportional to the magnitude of eDNA abundance (mean mtDNA copies) and are colored based on species identification from Sanger sequencing: red for alewife and blue for blueback herring. eDNA copy numbers reflect the initial number of mtDNA copies per 40 mL of water filtered.
(PNG)
