# Supplementary figures and images for: Environmental DNA analysis of river herring in Chesapeake Bay: A powerful tool for monitoring threatened keystone species
Source: PLoS One. 2018 Nov 1;13(11):e0205578. doi: 10.1371/journal.pone.0205578 (PMC6211659; doi:10.1371/journal.pone.0205578)

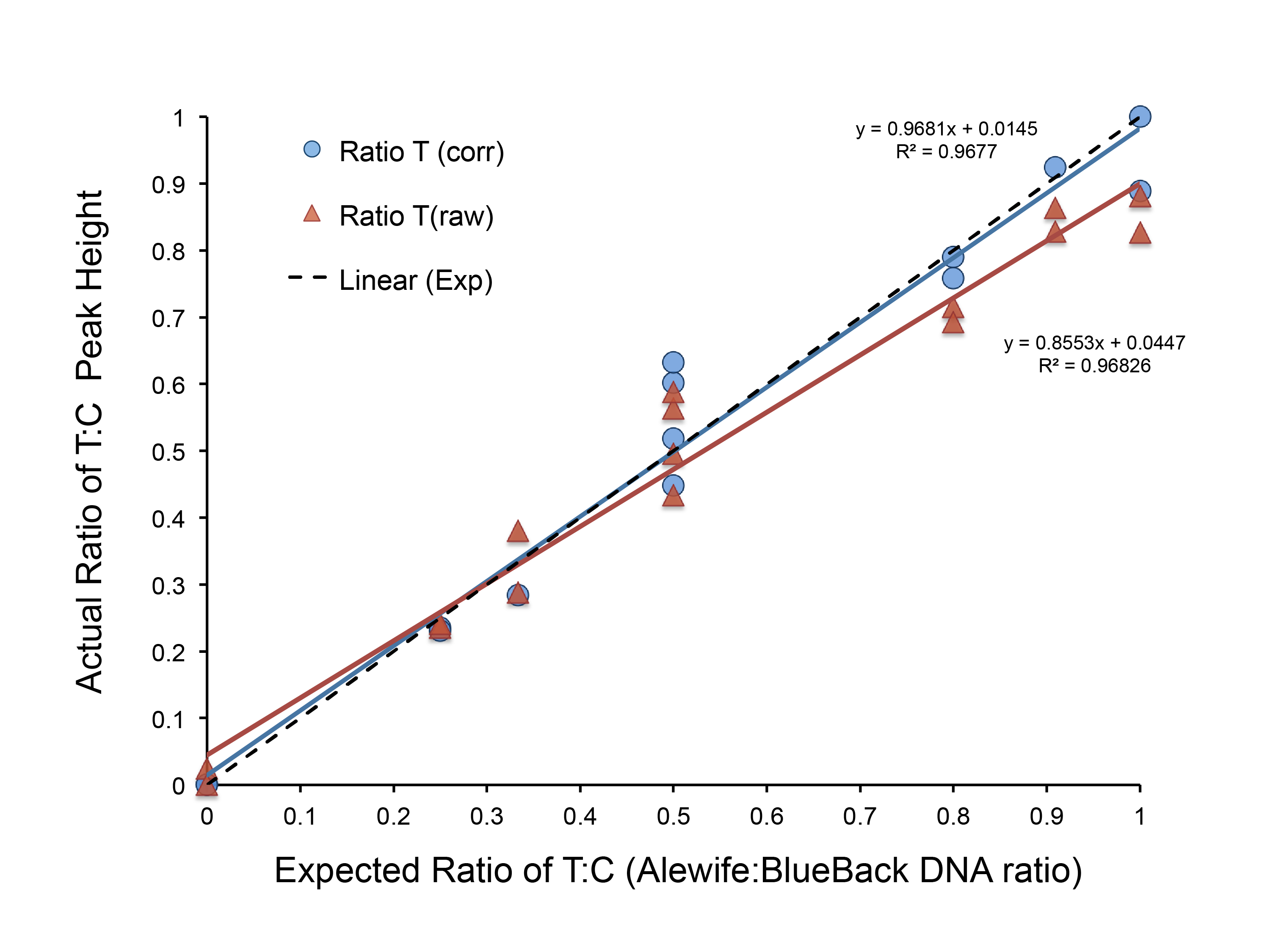

Supplement: S1 Fig — Plot of expected species DNA ratios (from 1:10 to 10:1 alewife:blueback DNA added to PCR) vs. observed DNA ratios inferred from peak height ratio analysis using QSVAnalyser at the diagnostic SNP at bp 104 (alewife = T, blueback = C) after sequencing the PCR amplicon. Ordinary least squares (OLS) regression lines are plotted for raw (red) and corrected (blue) peak height ratios compared to a 1:1 line (dashed) alongside OLS equations and R2 values (below the curves for the raw peak height ratios, above the curves for the corrected peak height ratios). (TIF) [file pone.0205578.s002.tif]

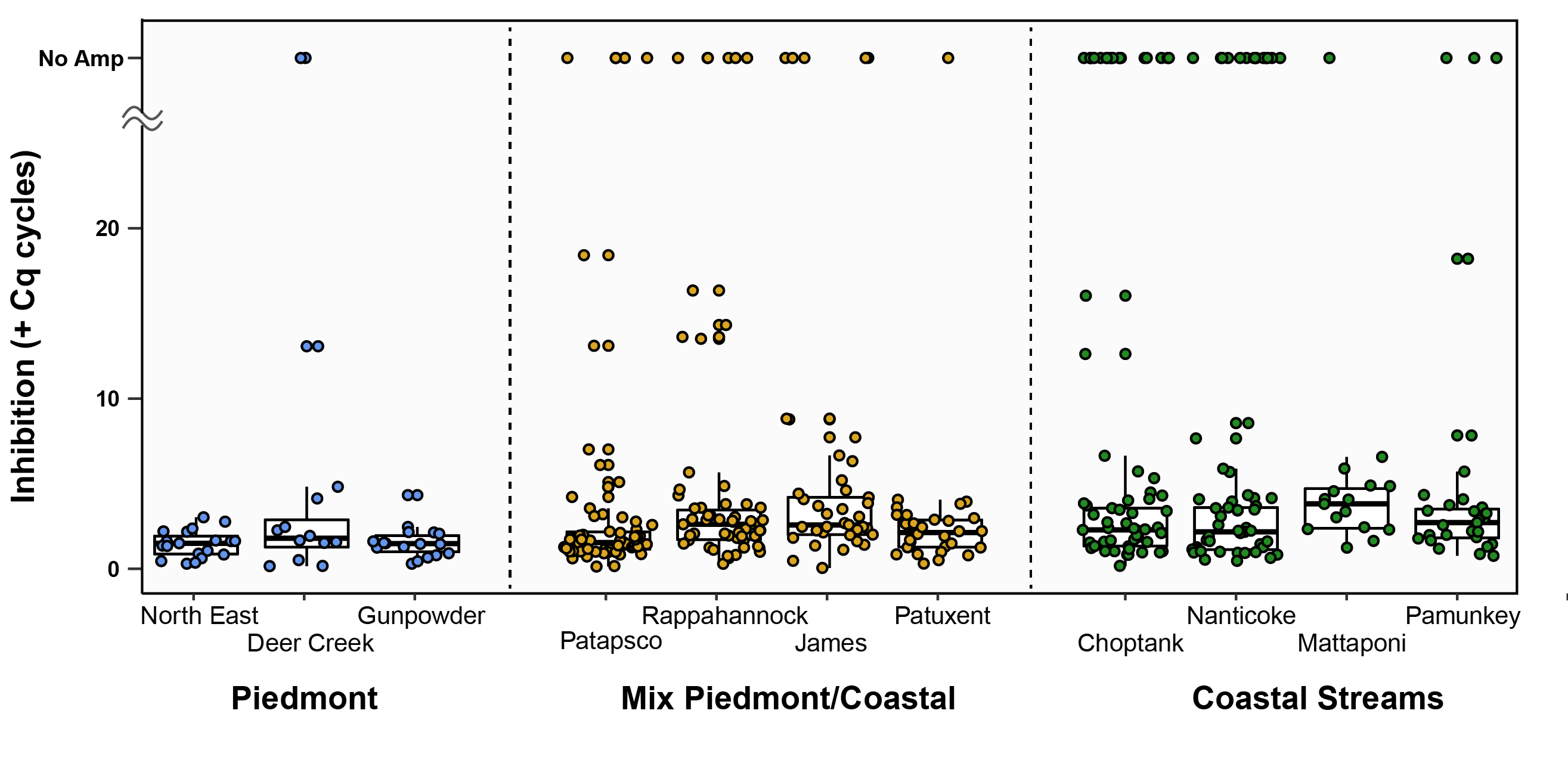

Supplement: S2 Fig — For each river or steam (grouped by type: piedmont, coastal, and mixed) inhibition is plotted as the number of additional qPCR cycles (Cq values) of the 300,000 copy oligo standard after spiking it with a given environmental sample. Note the break in the Y axis–samples that produced no amplification (complete inhibition) are plotted at ‘No Amp’. (TIF) [file pone.0205578.s003.tif]

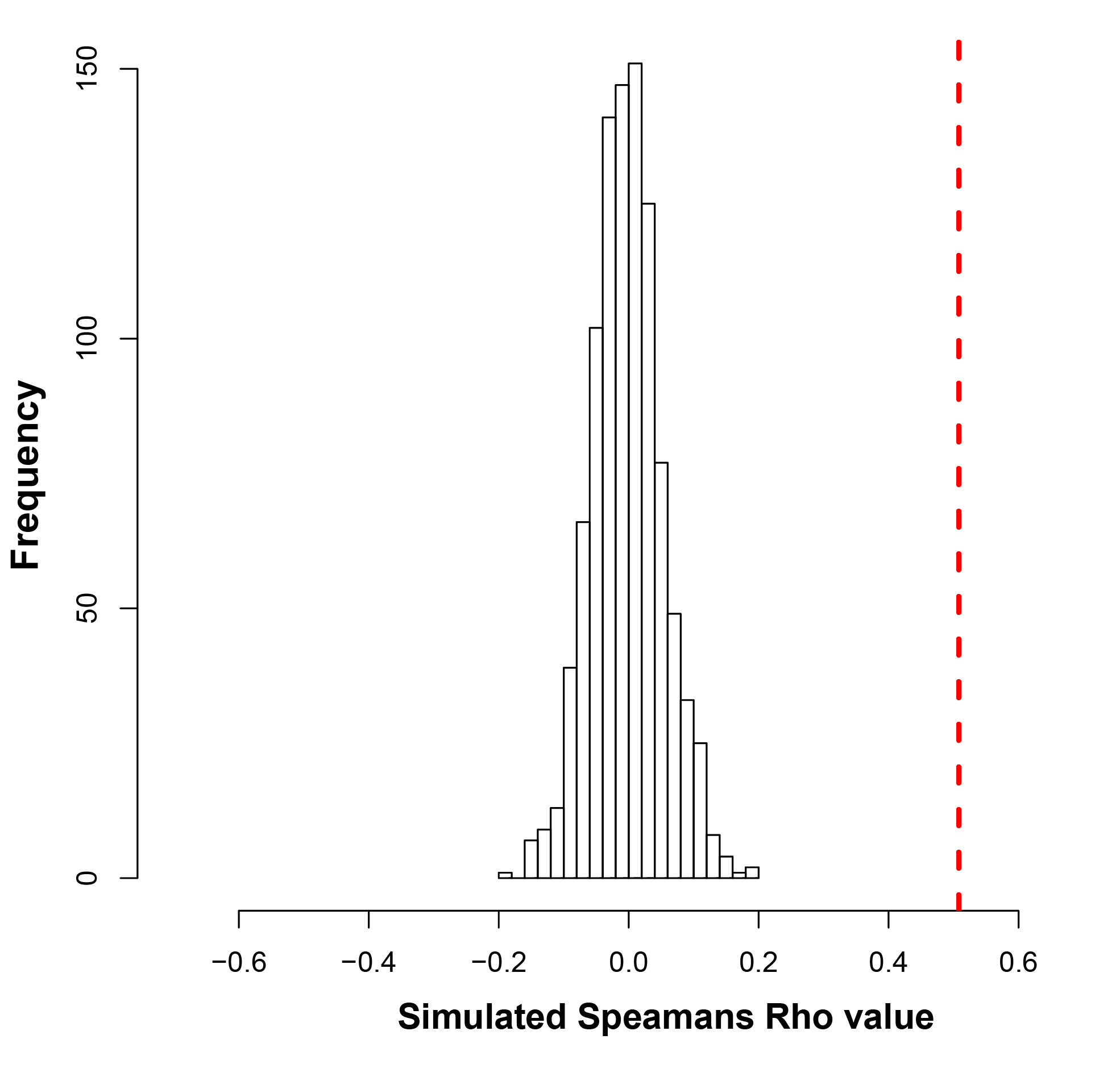

Supplement: S3 Fig — Histogram of Spearman’s Rho values from 1000 random permutations of the eDNA and ichthyoplankton datasets. The dashed red line shows the observed estimate of correlation between eDNA and Ichthyoplankton abundance datasets (Rho = 0.52). (TIF) [file pone.0205578.s004.tif]

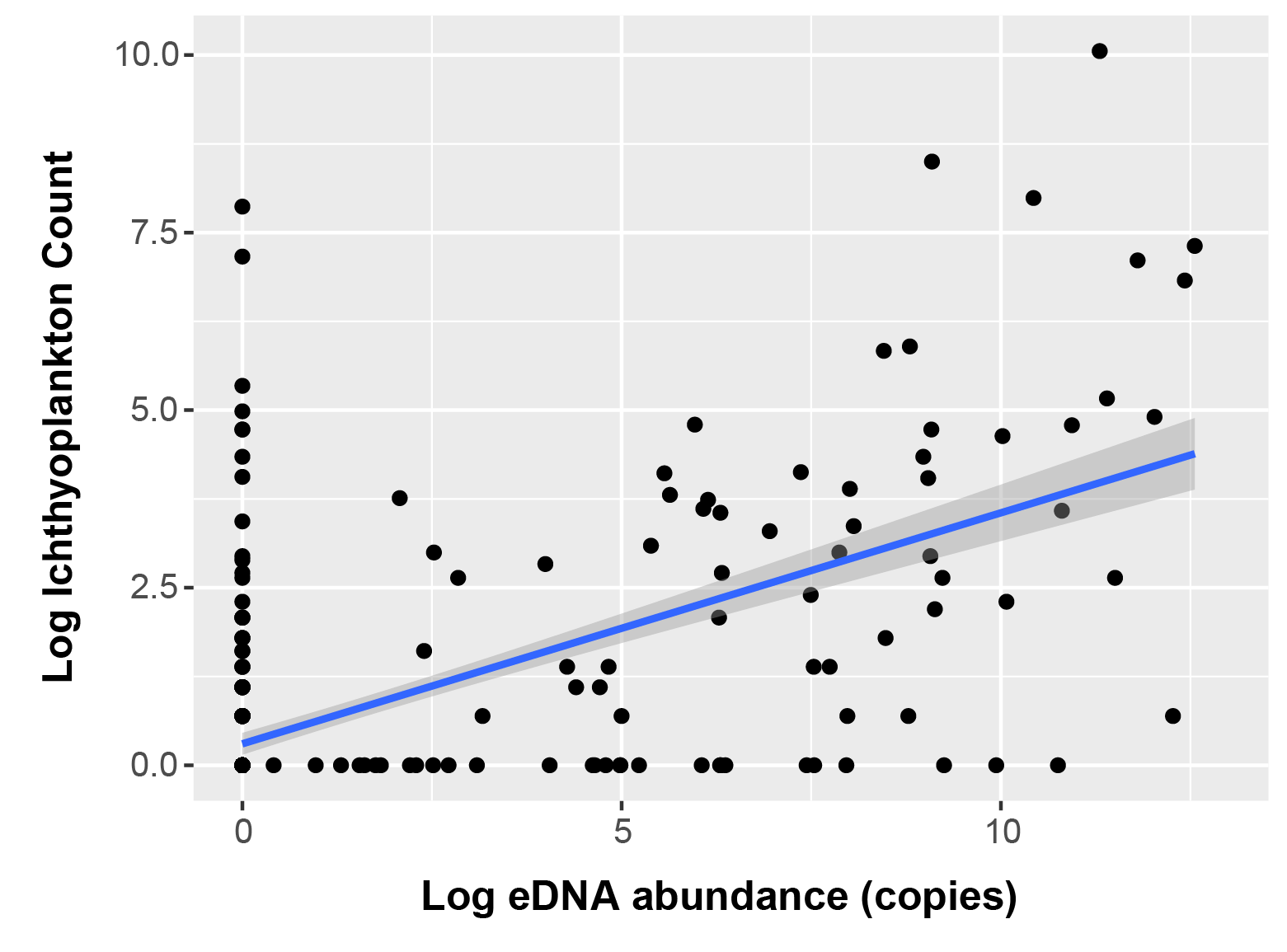

Supplement: S4 Fig — Data are plotted on a log-log scale. eDNA copy numbers reflect the initial number of mtDNA copies per 40 mL of water filtered. (TIF) [file pone.0205578.s005.tif]

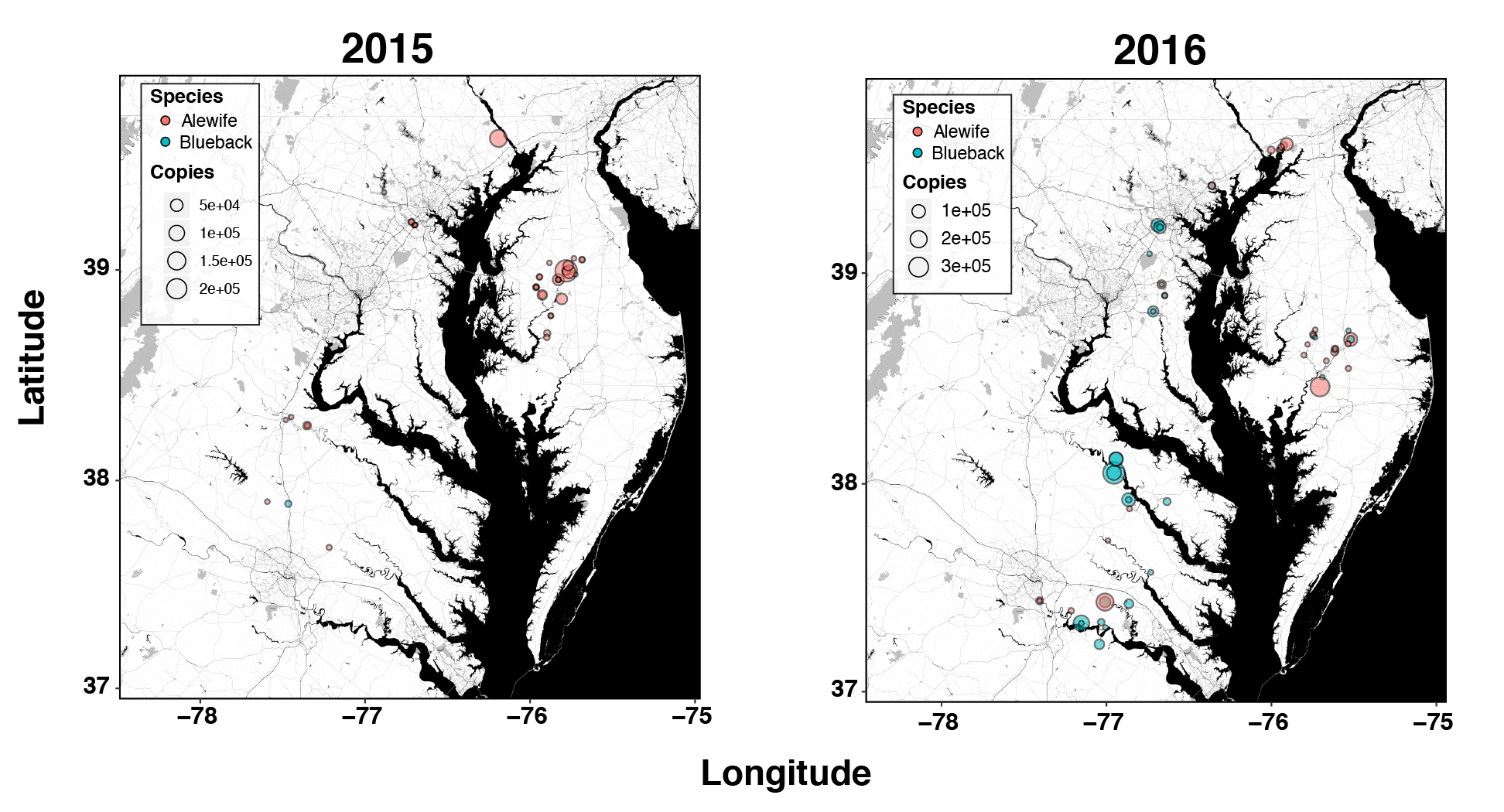

Supplement: S5 Fig — Size of data points (positive detections) are proportional to the magnitude of eDNA abundance (mean mtDNA copies) and are colored based on species identification from Sanger sequencing: red for alewife and blue for blueback herring. eDNA copy numbers reflect the initial number of mtDNA copies per 40 mL of water filtered. (PNG) [file pone.0205578.s006.png]
